# LARC methods: entering a new age of contraception and reproductive health

**DOI:** 10.1186/s40834-016-0011-8

**Published:** 2016-02-23

**Authors:** Donna Shoupe

**Affiliations:** University of Southern California, Los Angeles, California USA

## Introduction

**Fig. 1:**
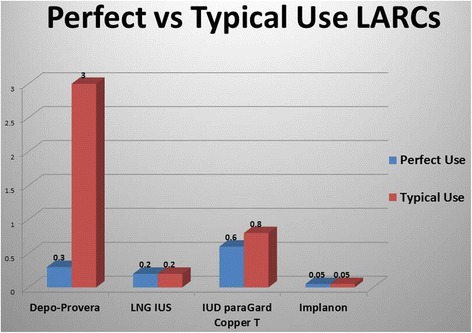
Perfect vs Typical Use LARCs

Although IUDs and implants have been around for a long time, their use has been severely hampered and almost extinguished for periods of time due to early design flaws, difficult insertion and removal demands, or by the unacceptable side effect profiles. It is all very different now. The currently available LARC methods are easy to use, safe, long lasting, quickly reversible and 20 times more effective than oral contraceptive pills [1–4] (Fig. 1). The LARC methods have high patient acceptability [5], have limited contraindications for use, and are often recommended, in some cases, due to their dramatically improved bleeding control. All of the LARC methods can be inserted right after delivery or abortion and following removal, fertility is rapidly restored. Although the LARC methods have a high up-front cost, most or all of these costs are often covered by a 3^rd^ party. In any case, compared to other options, they are highly cost-effective in the long-term [6–10].

Use of the LARC methods in the U.S. has traditionally trailed dramatically behind Europe [11], Asia and developing countries. Worldwide, the IUD is the currently the most popular means of reversible birth control in the world with 160 million users (2/3 of the users are in China) [12]. The good news is that as education and understanding of these new generations of LARC methods is getting to U.S. consumers, their popularity has had remarkable continual growth [13].

### Troubled history of long-acting reversible contraceptive methods

#### IUD history

The first IUD was reported from Poland in 1909. This intrauterine ring was modified in 1920-30 by a Germany scientist and separately by a Japanese physician in1934. In 1949 another German scientist who had immigrated to the US developed a stainless steel ring IUD.

The U.S. FDA first approved IUDs in 1968 eventually bringing the Lippes Loop, the Tatum-T, The Saf-T-Coil, Gynekoil and Copper 7 to the U.S. market. These IUDs were made with plastic, allowing the IUD to bend for insertion and then regain its shape. These IUDs also had a monofilament nylon string added to facilitate removal. The Lippes Loop, one of the most popular first generation IUDs, was introduced in the mid 1960’s. From 1960-1970, 12 million women worldwide had IUDs inserted including 3 million living in the US.

By 1970 there were over 17 IUDs in development worldwide. With the discovery that the addition of a copper band to the IUD increased effectiveness, a new generation of IUDs [Tatum-T and Copper-7] was introduced. Researchers also addressed the bleeding and cramping problems associated with copper containing IUDs with a new wave of technology directed at developing IUDs that released micro-dose progestin. By adding the hormone, these IUDs were noted to decrease cramping and bleeding by an average of 90 %.

But the popularity and use of all IUDs was soon to be in serious jeopardy, particularly in the U.S., as a new IUD with a design flaw was already gaining popularity in the U.S. market. The Dalkon Shield, designed with fins to resist expulsion, was introduced in 1968. These fins had the disadvantage of making removal difficult and a super-strong multifilament string was added. By 1974, after over 2.2 million had been sold in the US, A.H. Robins suspended their sales. Reportedly six women had died due to complications of the IUD and thousands more had suffered serious infections. Scientific work eventually discovered a serious design flaw in the multifilament string. Studies using scanning electron microscopy reported that all strings that had been removed from users showed deterioration of the outer sheath and bacterial contamination of the underlying multifilaments [14, 15]. This finding is the likely cause of ascending infection seen by so many Dalkon Shield users, resulted from contamination from either an ascending infection up the string or from direct contamination when an IUD was pulled into the endometrium as with an ongoing pregnancy [16, 17]. Even though the Dalkon Shield was the only IUD implicated, the ensuing debacle affected the reputation of all IUDs. By 1986, all but 1 IUD (Progestasert – manufactured until 2001) was pulled from the U.S. market. For 2 years the U.S. market for IUDs almost disappeared.

In 1988 GynoPharma introduced a new copper IUD, the T-380 A (ParaGard), to the U.S. market. This introduction started the rebound of IUD use in the U.S. A new hormonal IUD (Mirena) was approved by the FDA in 2000 and the smaller 3-year version, Skyla, was approved in 2013. 17 The most recent approval from the FDA was on March 2, 2015 for a new IUD that was designed with affordability in mind. Liletta is very similar to the Mirena although it is now only approved for 3 years of use. Ongoing studies should result in a longer approved use.

Due to efforts by public health professionals including the American College of Obstetrics and Gynecology and the Centers for Disease Control and Prevention (CDC), public opinion and use of IUDs has been steadily improving [18–20].

#### Implant history

Implants have also had their controversies and challenges. In 1990 the six rods implant (Norplant) was approved by the U.S. FDA. By 2002 Norplant was taken off the market due to limitations of product supplies, issues of coercion in women convicted of drug or child abuse, as well as issues related to difficult removals [21, 22]. For the next 4 years there were no implants available in the US. The return of contraceptive implants began in 2006 when the FDA approved the one rod implant system (Implanon). Clinical trials had shown that the average insertion time was only one minute and the average removal time was only 3 minutes. In 2010 Implanon was replaced with the next generation implant, Nexplanon. The newer implant featured an improved, easier to use inserter and the addition of a barium marker on the implant making it more detectable by imaging techniques.

The ongoing efforts of large numbers of public health professionals, national organizations, and healthcare providers to educate the public on the safety and efficacy of the LARC methods has benefited both IUDs and the implant [18–20, 23–28]. While the popularity of the implant is still lagging behind the rapid increases seen in IUD use, implant demand is steadily climbing.

### Updated guidelines now encourage the use of LARCs in all age groups

In 2009, LARC methods became first-line options when the American College of Obstetricians and Gynecologists recommended LARC methods for the majority of women [29]. Since then the growing support had been clear and wide-spread. The CDC Eligibility Criteria for Contraceptive Use recommends LARC methods for the majority of women who have their first menses, regardless of whether they have had any pregnancies [11]. The American Academy of Pediatrics recommends LARC methods for adolescents as “prevention is the cornerstone of pediatric practice” [20]. A recently published article in Pediatrics: the Official Journal of the American Academy of Pediatrics stated “We suggest that in response to the improvement in the effectiveness and safety of long-acting reversible contraceptive (LARC’s: i.e. IUD and implants ), pediatricians have a special opportunity to prevent unintended pregnancy” [30].

ACOG revised one of their practice guidelines on LARCs in 2012. The new guidelines recommended that sexually active adolescents at high risk for unintended pregnancy should be encouraged to consider LARCs [27]. In its Family Planning Handbook for Providers, WHO recommends the implants and IUDs for women with or without children of any age, including adolescents and women over 40 [28]. In an editorial in the Association for Reproductive Health Professional’s Contraceptive Journal, the authors decry the “outdated perceptions about appropriate patient candidates for LARC among health care providers continue to negatively impact their use.” An emerging body of research has disproved a number of contraindications to IUC use. Specifically women of any age or parity and those who are postpartum or post first or second trimester abortion are eligible for IUC [31].

### Efforts to increase LARC use in young women

Public health professionals have recently been developing programs to increase knowledge and use of LARC methods among young women. The Contraceptive CHOICE project was conducted by researchers at Washington University in Saint Louis. These researchers noted the high rates of unintended pregnancies and abortions in the U.S. including 273,105 babies born to teens 15–19 in 2013 [32]. Their goal was to eliminate cost barriers and increase patient access to LARC methods particularly in young women in their region.

The CHOICE researchers developed a standardized script that included tiered counseling (most effective methods first) of all reversible methods. The “menu of options” listed hormonal IUD first, followed by the copper IUD and implant, then injections, pills, patch, vaginal ring, condoms and lastly emergency contraception. In their overall cohort 75 % of patients chose LARC methods while 72 % of teens opted for LARC methods. The overall 12 month continuation rate of the LARC methods was 86 % (with the LNG-IUS with the highest continuation rate 87.5 %). All of the other methods had 56–49 % twelve month continuation rate. The satisfaction rate in the overall cohort was 78–85 % in the LARC methods and between 54–44 % for the other method. In the 14–19 year old group, satisfaction was between 74–77 % for the LARC methods and between 31–46 % for the other methods [33, 34].

These researchers projected that if the CHOICE model was adopted nationally among all sexually active teens in the U.S., the pregnancy rate of 67.8 per 1,000 teens (in 2008) [33] could be reduced to 29.6. [33, 34] Confirmation of this assertion was a recent paper showing reductions in teenage pregnancy and abortion rates in England as LARC usage increases [35].

### Overall use

According to the Guttmacher Institute, the use of LARC methods has in the U.S. has jumped to 12 %, “the highest ever recorded” [36]. While the overall use of contraceptive use in reproductive-aged women has not changed, the newer data shows an ongoing shift towards the LARC methods. For comparison, in 2002 only 2.4 % of contraceptive users relied on LARC methods and in 2007, this number was 8.5 %. The top methods of choice are the OCPS (26 %) followed by the condom (15 %) and now LARC methods (12 %) [36].

The good news behind this upward trend of LARC use is the accompanying downward slope of unwanted pregnancies in the U.S. and 13 % decline in abortions between 2008 and 2011 [36]. CDC data reporting increased long-term use of LARC methods is coupled with data showing declines in overall births and abortions. The Affordable Care Act (ACA) that guarantees coverage of contraceptives for most women, including LARC methods, had been instrumental in the dramatic increase in LARC use. Protecting this funding source is a priority if this downward trend in unintended pregnancy rates in the US is to continue. Other areas of priority include Title X, Medicaid, and extension of the ACA into all states [36, 37].

## Worldwide use

Worldwide use of contraceptives shows uneven progress. Most developing countries have generally seen consistent growth of contraceptive use, particularly the more effective methods, while in others, such as Nepal and Jordan, the use has leveled off or even fallen slightly. In many of the less and least developed countries there are large unmet needs for family planning [38, 39].

It is interesting, however, to compare the use of modern methods, particularly LARC use in countries around the world (compiled in 2013). There is a great deal of variation of contraceptive use worldwide with high unmet needs among young unmarried women especially in less developed poorer countries.

### Effectiveness

LARC methods are around 20 times more effective than any other type of reversible birth control excluding the DMPA injection [1–3]. In a 2012 study of over 7400 participants, the failure rates in participants using oral contraceptive pills, birth control patch, or the vaginal ring was 17-20 times higher than the risk of those using LARC methods. For those under 21 using the pills, patch or ring, the risk of failure was almost twice as high as the older participants. But rates of unintended pregnancy regardless of age remained low in the LARC (and depo-provera group) [1].

### Contraindications

For common conditions, there are very few contraindications to LARC methods. ACOG endorses the US Medical Eligibility Criteria from the CDC which report the following contraindications. Contraindications to insertion of any IUD is the presence of cervicitis, current chlamydial infection or gonorrhea, distorted uterine cavity, current PID, cervical cancer awaiting treatment, suspicious (for serious disease) unexplained vaginal bleeding, puerperal sepsis, pregnancy, gestational trophoblastic disease, AIDS (category 3 -risks may outweigh benefits), complicated organ transplant (category 3), hepatocellular adenoma or malignant liver tumor (3) [40].

Contraindications for the hormonal IUD are history or current breast cancer, severe decompensated cirrhosis, SLE with positive or unknown antiphospholipid antibodies (category 3). Contraindication for copper IUD insertion is severe thrombocytopenia (category 3), [40]

Implants also have few contraindications, including history or current breast cancer, severe decompensated cirrhosis, suspicious for serious cause unexplained vaginal bleeding (category 3 , SLE with positive or unknown antiphospholipid antibodies (category 3), hepatocellular adenoma or malignant liver tumor (category 3) The bleeding pattern after implant insertion is most commonly less bleeding or similar bleeding but in a small percentage of women, heavier, unpredictable bleeding may occur [40].

Importantly, IUDs and Implant can be used in the following conditions [40]:

- Insertion in presence of inflammatory bowel disease,
- current history of ischemic heart disease
- multiple risk factors for arterial CV disease
- ovarian cancer
- past history of PID
- immediate postpartum
- severe dysmenorrhea
- vaginitis
- stroke
- valvular heart disease
- fibroids
- anticoagulation therapy
- most antiretroviral therapy
- antimicrobial therapy
- sickle cell disease
- DVT/PE established on anticoagulant therapy for 3 months
- family history DVT/PE
- major surgery
- depressive disorders
- diabetes with or without vascular disease
- migraines with or without aura
- HIV infected or high risk for getting HIV
- The copper IUD can be used in women after breast cancer

### Counseling: risks and benefits

The currently available LARC methods are safe, easy to use, long lasting, quickly reversible and 20 times more effective than oral contraceptive pills [1–4]. The LARC methods have high patient acceptability, 5 and generally have few contraindications for use [40]. The hormonal IUD is associated with dramatic reductions in bleeding, often amenorrhea, reduced cramping and reduced pain associated with endometriosis. All of the LARC methods can be inserted right after delivery or abortion and following removal, fertility is rapidly restored. Although the LARC methods have a high up-front cost, most or all of these costs are often covered by a 3^rd^ party. In any case, compared to other options, they are highly cost-effective in the long-term [6–10].

Importantly, modern IUDs do not carry a risk of pelvic infection after the first 20 days after insertion [2–41]. There is a very small risk of uterine perforation (much less common than in the past due to advanced IUD designs), or expulsion. Heavier bleeding is a side effect of the copper-IUD while a dramatic reduction in menses is a benefit of the hormonal IUD. A small percentage of women with the hormonal IUD may experience progestin side effects such a mood changes or increased acne [42].

Contraceptive implants generally reduce bleeding and a complete cessation of menstrual flow can occur. However, some women may experience irregular and/or increased bleeding. Progestin side effects are not common with the implant but may include increased acne or mood changes [43, 44].

### Future directions: identifying barriers and increasing LARC Use

The preceding discussion has provided evidence that LARC methods are safe, reversible, have high patient acceptability, and very few contraindications. Newer generation LARCs, such as the levonorgestrel containing IUDs, also have non contraceptive benefits. Perhaps most importantly, these methods are 20 times more effective than oral contraceptive pills [1–4]. Despite the numerous demonstrated advantages of LARCs, uptake remains low in the U.S. and many countries throughout the world (Table 1). In the following section, Future Directions, we will examine the barriers to LARC use and identify current and potential strategies for increasing LARC uptake and adherence.

**Table 1:** Modern Contraceptive Use Worldwide

	Married Women using Modern Methods	Married Women using LARC Methods
WORLD	57 %	13 %
Africa	27 %	5 %
South America	71 %	5 %
Asia	61 %	17 %
Asia excluding China	48 %	6 %
Eastern Europe	53 %	17–25 % (excluding Russia)
Western Europe	67–74 %	8–22 %
Northern Europe	82 %	13 %
Australia	68 %	4 %
Vietnam	60 %	32 %
North Korea	58 %	42 %
South Korea	70 %	12 %
UK	84 %	11 %
China	84 %	40 %
Syria	33 %	20 %
Egypt	57 %	36 %
Cuba	73 %	25 %
Canada	72 %	1 %
USA	73 %	6 %

Adapted from Family Planning Worldwide 2013 Ref 38

There are multiple barriers to LARC use [45, 46]. Some of these barriers are global in nature and some of them are unique to specific populations such as post-partum women, minority and low-income women or geographic locations which are resource limited [47, 48]. A knowledge deficit about LARCs exists among many healthcare professionals. This lack of education and training may result in significant barriers to LARC access that are linked to the provider or pharmacist [49]. In a recent American College of Obstetricians and Gynecologists Practice Bulletin some of the primary barriers to LARC use cited included cost, provider experience, and interest, as well as patient interest [50] (Table 2).

**Table 2:**
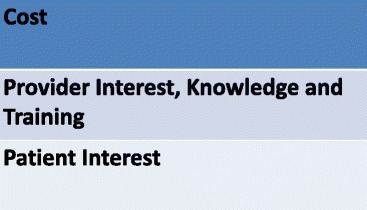
Barriers to LARC Use

### Cost

Women living in poverty have higher rates of unintended pregnancy as well as abortion [51, 52]. The CHOICE study clearly demonstrated that unintended pregnancy rates can be dramatically reduced by eliminating financial barriers. When cost is not a factor this study found that 75 % of eligible women chose a LARC method [53]. Although multiple studies have shown that investment in contraception leads to significant cost-savings, access to LARC methods by poor women remains challenging. In a frequently cited publication by Trussell et al, a decision model was used to evaluate cost of contraceptive methods from a payer perspective. The study results demonstrated that the use of any contraceptive method resulted in cost-savings compared to use of no method. This included LARCs, which despite a higher upfront cost, are more cost effective as a result of their high efficacy [54].

Foster et al provided additional support for the findings of Trussell and colleagues in their study of a California Medicaid amendment. The California Family Planning, Access, Care, and Treatment (Family PACT), is a Medicaid State Plan Amendment that serves more than 1.8 million clients per year at or below 200 % of the federal poverty level. The authors found that public cost-savings for each dollar spent on contraception ranged from $1.58 for barrier methods to approximately $5 for LARC methods. Short-term hormonal methods and DMPA demonstrated intermediate cost-savings resulting in cost savings from $2.12 for the patch to $4.00 with DMPA, Most significantly this study demonstrated use of LARC methods is cost-effective even if methods are not used for their full durations of efficacy [55].

### Provider interest, knowledge and training

Multiple authors have shown that provider beliefs and practices pose a significant barrier to the widespread use of LARCs. An ACOG supported survey of fellows demonstrated that even when providers consider it appropriate to provide LARCs, a much smaller percentage of providers actually offer them in practice. The vast majority of obstetrician gynecologists offer IUDs (95.8 %), but a majority require two or more visits, which is a potential barrier to more wide spread use. Although 67 % of respondents felt it was appropriate to offer IUDs after spontaneous or therapeutic abortion, only 10.9 % offered them to patients. Nearly half (43.5 %) of fellows surveyed thought it was appropriate to offer IUDs in the immediate postpartum period, only 7.2 % offered this method to patients (Table 3) [56].

**Table 3:**
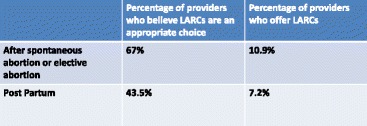
Provider Beliefs and Practices

Education and training is a significant predictor of the use of LARCs. A lack of training, particularly training during residency, is an important barrier to the use of these contraceptives. Nearly a third of obstetrician gynecologists who were surveyed reported a lack of insertion training as a barrier to the use of contraceptive implants. Although a large majority (92 %), reported IUD insertion training during residency, only 50 % reported training on implants. Continuing education within the past two years was, in fact, the best predictor of implant provision. This is critically important because as the authors point out, “clinicians are gatekeepers of LARC services” [56].

### Patient interest

Patient interest is a critical factor in increasing LARC uptake. The CHOICE study provided clear evidence that patient interest in LARCs can be dramatically increased with education and reduction of economic barriers. 1 When obstetrician gynecologists were surveyed about the use of contraceptive implants, 46 % cited lack of patient interest as the reason for not offering this method [56]. In a study of predictors of LARC use among unmarried young adults, women with high IUD knowledge were six times more likely to be current LARC users (OR 6.3). High IUD knowledge was the strongest predictor of LARC use in the adjusted model [57].

Age is also an important predictor of interest in LARCs. Women ages 18–19 are less likely to report current LARC use compared with 25–29 year olds (OR 0.1 confidence interval 0.02-0.4) [57]. This statistic highlights the important role of adolescent health care providers in encouraging the use of LARCs in younger women. In a study of adolescent health care providers residency training in obstetrics and gynecology or family medicine was the strongest predictor of LARC provision, especially IUDs [58]. Education and training in LARC procedures appears to be an important factor in the provider’s role in encouraging interest in LARCs. Provider knowledge appears to be a prerequisite for patient education about LARCs [58].

### Strategies for increasing LARC use

The increased uptake of LARCs has the potential to be an important strategy to decrease the unintended pregnancy rate. Multiple clinical studies have indicated that enhanced access to these methods has a dramatic impact [1, 2, 7]. In the following section strategies to increase LARC access and use will be discussed.

### Reducing cost

An important consideration in evaluating the cost of LARCs is documentation of the cost savings associated with these methods, and not just the upfront cost of the contraceptive. In a recent article in the N.Y. Times the author examined the remarkable success of the Colorado state effort to reduce teen pregnancies. The article reported that the state health department estimated that every dollar spent on the long-acting birth control initiative saved $5.85 for the state’s Medicaid program, which covers more than three-quarters of teenage pregnancies and births [7]. Multiple scientific manuscripts have also documented the cost-effectiveness of these methods [54, 55]. Cost analysis studies that have assessed the cost benefit of contraception have nearly universally found that contraception, particularly highly effective contraception, is cost effective. Studies by the Guttmacher Institute found that for every dollar spent on family planning services, $1.30 would be saved on maternal and newborn health care [59]. It is critical that part of the advocacy process for LARCs includes education of those involved in healthcare related legislation and healthcare services.

Another important strategy is the reduction of cost through large scale purchases. The Department of Defense healthcare services and large HMOs such as Kaiser Permante, as well as non-governmental organizations (NGOs) such as the Bill and Melinda Gates Foundation illustrate the potential to reduce LARC costs when these drugs and devices are bought in bulk. More than 200 million patients in the U.S. receive their health care through managed care organizations. This statistics illustrates the important role of managed care pharmacies in securing lower drug and device prices. HMOs have the ability to negotiate volume discounts, which results in lower drug expenditures and greater profits for the HMOs. These cost savings can be achieved in a number of ways including, discount off invoice or bulk discounts, and rebate agreements [60]. Prescription drug coverage is one of the ten essential benefits required by the Affordable Health Care Act. Because the ACA makes drug coverage a core part of health insurance, it eliminates the insurers’ ability to tack on a prescription drug benefit plan to a health care plan at an additional cost. As a part of preventative care, prescription birth control is now free if generic, and available through a co-pay if brand name [61].

### Increasing provider interest, knowledge and training

Although the majority of gynecologists have training in IUD insertion, a smaller percentage have training in implant insertion, and many who have training do not have adequate knowledge of patient eligibility [62, 63]. The American College of Obstetricians and Gynecologists (ACOG) has created a number of programs to enhance provider education. ACOG provides a list of LARC Clinical Training Opportunities as a LARC Slide Set and a LARC for Adolescents Slide Set. The organization also provides a list of Family Planning Speakers with special expertise in LARCs [62].

Family physicians also provide a great deal of the contraceptive counseling and provision in the U.S. Although the vast majority of family physicians believe patients are receptive to learning about IUDs, one study found that less than half offer counseling or the method. Both gynecologists and family physicians were found to have inadequate knowledge of IUD eligibility as gauged by the CDC and Prevention Medical Eligibility Criteria for contraception. Family physicians did report an interest in updating contraceptive skills. There is clearly an opportunity to increase LARC uptake through training and education of physician providers [63].

Nurse practitioners are often the primary providers of contraception for a number of women. It is important that women’s health nurse practitioners are trained not only in contraceptive counseling, but also in IUD insertion. In a study of LARC counseling and provision by women’s health nurse practitioners, two thirds (66 %), were trained in IUD insertion. This compared to only 12 % of primary care nurse practitioners. Contraceptive counseling, however, included IUDs in 43 % of cases. Nurse practitioners were found to use overly restrictive patient eligibility criteria which was inconsistent with the CDC guidelines. Both insertion training and knowledge of patient eligibility were significantly associated with IUD provision. Contraceptive implant provision was also low, with only 42 % of NPs in women’s health and 10 % of primary care NPs providing implants to their patients [64]. Increasing training and education for nurse practitioners who provide contraception will play a critical role in increasing LARC usage as healthcare reforms focused on affordable primary care are put into practice.

Pharmacists are becoming important providers of contraceptive services, but they are often not considered as advocates for increasing LARC uptake. For a number of years pharmacists have provided emergency contraception. Now that emergency contraception is available over the counter, patients are more likely to look to pharmacists for medical advice. Women who seek emergency contraception provide an opportunity for pharmacists to provide LARC education and referrals. Pharmacists, as providers of emergency contraception, are well positioned to intervene with patients at high risk for unintended pregnancy. Both the American College of Clinical Pharmacy (ACCP) and the Women’s Health Practice and Research Network (PRN) advocate an expanded role for pharmacists in advocating for and facilitating the use of LARC methods [65].

### Increasing patient interest

The CHOICE study is a model of increasing patient interest and LARC use through patient education. Through the development of a standardized script that included tiered counseling [most effective methods first] of all reversible methods, LARC usage in this study was markedly increased. The investigators found 75 % of patients chose LARC methods while 72 % of teens opted for LARC methods. The overall 12 month continuation rate of the LARC methods was very high at 86 %. The satisfaction rate was also high at 78–85 % in the LARC methods compared to 54–44 % for the other methods. Among adolescents the satisfaction rate was between 74–77 % for the LARC methods and between 31–46 % for the other methods [33, 34].

To successfully increase LARC uptake outside the setting of a clinical trial, other strategies for increasing patient education and interest must be identified. In a study from the United Kingdom examining the etiology of low uptake of LARCs, the authors identified dissemination of information in multiple venues such as health centers, schools, peer education, as well as the use of multiple media forms. Increasing primary healthcare nurses’ role in contraceptive counseling and provision was also considered an important strategy [65].

## Conclusions

The safety, convenience and ability of LARC use to impact high rates of unintended pregnancy have been documented in numerous clinical trials as well as in health care settings that provide increased access to these methods [1, 2, 7]. The challenges to increase LARC uptake are many and include decreasing costs, insuring easy access to training, providing increased patient knowledge and encouraging patient interest. Overcoming these barriers requires a multifaceted strategy which involves patients, providers, healthcare administrators, legislators and the community.
